# Purification and Structural Characterization of Sulfated Polysaccharides Derived from Brown Algae, *Sargassum binderi*: Inhibitory Mechanism of iNOS and COX-2 Pathway Interaction

**DOI:** 10.3390/antiox10060822

**Published:** 2021-05-21

**Authors:** Jun-Geon Je, Hyo-Geun Lee, Kurukulasuriya H. N. Fernando, You-Jin Jeon, Bomi Ryu

**Affiliations:** 1 Department of Marine Life Sciences, Jeju National University, Jeju 63243, Korea; wpwnsrjs@naver.com (J.-G.J.); hyogeunlee92@gmail.com (H.-G.L.); 2 Marine Science Institute, Jeju National University, Jeju 63243, Korea; hiruninfdo@gmail.com

**Keywords:** *Sargassum binderi*, sulfated polysaccharide, inflammation, macrophages, zebrafish

## Abstract

Among the components derived from brown algae, anionic sulfated polysaccharides, which contain sulfated fucose as the major monosaccharide, exert significant biological activities. In this study, we purified and structurally characterized sulfated polysaccharides from brown algae, *Sargassum binderi* (*S. binderi*; SBPs), and evaluated their biological activity in vitro and in vivo. The SBPs were separated based on their charges and their biophysical properties were investigated according to their functional groups, structural features, and molecular weights using FTIR, NMR, and MALS. Among all the SBPs, Fraction 4 (SBP-F4), with an average molecular weight of 2.867 × 10^5^ g/mol, had the highest polysaccharide and sulfate contents (75.15 ± 0.25% and 24.08 ± 0.18%, respectively). The biological activities of SBP-F4 were investigated further in vitro and in vivo. Our results showed that SBP-F4 significantly suppressed the expression of inducible nitric oxide synthase (iNOS) and cyclooxygenase-2 (COX-2) proteins in LPS-activated macrophages. Moreover, in the LPS-treated zebrafish model, a significant decrease in cell death and NO production was observed. Collectively, these results show that SBPs not only exert protective effects against LPS-induced cytotoxicity but also inhibit the activation and anti-inflammatory activity of macrophages. Therefore, polysaccharides derived from *S. binderi* are potential anti-inflammatory agents for use in clinical settings.

## 1. Introduction

Brown algae are not only used as food, but have also been studied as a source of healthy functional foods and metabolites with medicinal properties [1,2]. Brown algae are a rich source of biologically active components such as polysaccharides, proteins, fatty acids, vitamins, minerals, and dietary fiber [3]. In addition, brown-algae-derived polysaccharides exhibit interesting structural features and diverse physiological activities [4].

Biologically derived polysaccharides have the advantages of nontoxicity, biodegradability, and biocompatibility, which makes them ideal candidates for widespread use [5]. Polysaccharides isolated from marine algae, especially fucose-rich polysaccharides isolated from brown algae, exert a variety of effects, such as antioxidant [6], anti-inflammatory [7], antimicrobial [8], and anticancer [9] effects. However, high-molecular-weight polysaccharides show limited biological activity due to their large molecular weight, high viscosity, and complex structures, which hinder their ability to enter cells or bind to receptors [10]. Therefore, prior studies have focused on isolating low-molecular-weight polysaccharides and homogenizing polysaccharides [11].

Sulfated polysaccharides have an average molecular weight of 20,000–200,000 Da, and are mainly found in various species of brown algae such as *Fucus vesiculosus*, *Ascophyllum nodosum*, and *Undaria pinnatifida* [12,13,14,15]. Fucoidan, composed of fucose, also includes other saccharides such as glucose, galactose, xylose, mannose, and uronic acid in varying proportions [16]. The content and monosaccharide composition of fucoidan in various algae depend on several external factors such as environmental conditions, harvesting period, and extraction and purification method [17,18]. As a result, fucoidan exhibits differential bioactivity depending on its constituent monosaccharides; moreover, fucoidan shows anticancer [15], antioxidant [19], and anti-inflammatory activities [20] depending on the fucose content. Therefore, the fucose content of polysaccharides is believed to affect other physiological activities as well.

*Sargassum* species are distributed worldwide. Among them, *Sargassum binderi* is found in the Indian Ocean, mostly in Malaysia [21], Sri Lanka [22], and Binuangeun [23]. A previous study reported that fucosterol isolated from *S. binderi* exerts anti-inflammatory activity in human lung epithelial cells [24]. Similarly, other *S. binderi* extracts showed antibacterial activity against human pathogenic bacteria [25], and toxicology studies based on these extracts showed that *S. binderi* could be used in the preparation of functional foods [26]. However structural and monosaccharide content characterization of fucoidans isolated from *S. binderi* remains unexplored [27]. Thus, the physical and chemical properties of sulfated polysaccharides isolated and purified from *S. binderi* need to be elucidated.

Although several studies have evaluated the bioactivity of fucoidan in vivo and in vitro, none of the studies investigated the biological effects of fucoidans isolated from *S. binderi*. Therefore, it is necessary to elucidate the fucose composition of the purified polysaccharides isolated from *S. binderi* and study their effect on biological activities, including anti-inflammatory activities.

In this study, *S. binderi* was hydrolyzed using Celluclast, and SBPs were obtained from the hydrolyzed product. As DEAE-cellulose has been extensively used to obtain fucose-rich polysaccharides [18,28,29], SBPs was purified using a DEAE-cellulose column and anion-exchange chromatography. We determined the chemical composition of the purified fraction (SBP-F1-4) and investigated its biological effects using LPS-activated macrophages (RAW 264.7) and a zebrafish model. This study elucidated the chemical components of fucose isolated from *S. binderi* and revealed the physical properties of sulfated polysaccharide fraction. Our study outcomes suggest SBPs as candidate anti-inflammatory agents for future use in clinical settings.

## 2. Materials and Methods

### 2.1. Materials

The brown algae *S. binderi* used in the experiment were collected from the Hikkaduwa southern coast of Sri Lanka in July 2019. To prepare the algal extracts, Celluclast was obtained from Novo Co. (Novozyme Nordisk, Bagsvaerd, Denmark); Dulbecco’s modified Eagle medium (DMEM), fetal bovine serum (FBS), penicillin–streptomycin, and trypsin-EDTA were purchased from Gibco/BRL (Burlington, ON, Canada) for cell-based experiments; and 3-(4,5-dimethylthiazol-2-yl)-2,5-diphenyltetrazolium bromide (MTT), dimethyl sulfoxide (DMSO), 4-amino-5-methylamino-2′,7′-difluorofluorescein diacetate (DAF-FM-DA), 3,6-bis(dimethylamino)acridine hydrochloride (acridine orange), and fucoidan for preparing polysaccharide standards were purchased from Sigma (St. Louis, MO, USA). Ultrapure water procured from the institutional water purification unit (Milli-Q synthesis Elix-10, Millipore Corp., Darmstadt, Germany) was used in this study. All chemicals and reagents used were of analytical grade.

### 2.2. Preparation of Polysaccharide

Briefly, *S. binderi* (SB) was washed with tap water more than three times to remove salt, sand, and other impurities. SB was washed carefully with fresh water and freeze-dried. The dried SB was ground and sieved through a 50 μm mesh. Polysaccharide extract was prepared by enzymatically hydrolyzing SB powder with Celluclast using a previously reported method [30], with slight modifications. The SB powder (100 g) was homogenized with 1 L distilled water, and the pH was adjusted to 4.0 using 1 N HCl. Subsequently, the homogenized mixture was mixed with 1 mL enzyme, and the enzymatic reaction was incubated for 24 h in an incubator set at 50 °C. The enzymatic reaction was stopped by incubating the reaction mixture in a 100 °C water bath for 10 min. The pH of the hydrolysate was adjusted to 7.0, with 1 N NaOH, and a 3-fold volume of 95% EtOH was added to precipitate the polysaccharides for 12 h at 4 °C. Finally, the precipitate was collected by centrifugation at 12,000× *g* for 20 min at 4 °C, and SB Celluclast hydrolysate was obtained by lyophilization. The SBPs from the hydrolysate was obtained by adding 95% ethanol to precipitate the polysaccharides.

### 2.3. Polysaccharide Separation by Anion-Exchange Chromatography

The anion-exchange chromatography using a DEAE-cellulose column was performed as reported earlier [31]. The DEAE-cellulose column was equilibrated with 50 mM sodium acetate (pH 5.0) and washed with the same buffer containing 50 mM NaCl. The elution was carried out at a flow rate of 20 mL/h with a linear gradient of 0–2000 mM NaCl containing 50 mM sodium acetate (pH 5.0). The polysaccharide content in each tube was measured using the phenol-sulfuric acid method, as described previously [32] using a standard curve with glucose (Sigma, St. Louis, MO, USA).

### 2.4. Characterization of Polysaccharides

#### 2.4.1. FTIR Analysis

The FTIR spectra of the SBPs were analyzed using an FTIR spectrometer (NicoletTM 6700; Thermo Scientific, Madison, WI, USA). Ten milligrams of powder was mixed with KBr and cast into pellets. The wavenumber range of 400–2000 cm^−1^ of the FTIR spectra, covering the fingerprint region of polysaccharides, was used to characterize the structural properties of the polysaccharides.

#### 2.4.2. NMR Analysis

For NMR analysis, the dialyzed SBP-F4 was lyophilized and deuterium was exchanged with deuterium oxide (D_2_O). The SBP-F4 dissolved in D_2_O was analyzed via ^1^H and 2D NMR spectra using a Bruker 800 MHz spectrometer at Korea Basic Science Institute (KBSI) in Ochang, South Korea.

#### 2.4.3. Monosaccharide Composition Analysis

The monosaccharide composition was determined by LC (Bio-LC system, Dionex, Sunnyvale, CA, USA) coupled with high-performance anion-exchange chromatography (HPAE-PAD) using a CarboPac^TM^ PA1 column (4.5 mm × 50 mm) [31]. The monosaccharides were detected under optimal isolation conditions (eluent: 18 mM NaOH/200 mM NaOH; flow rate: 1 mL/min; injection volume: 20 μL) at the Carbohydrate Bioproduct Research Center in Seoul, South Korea.

#### 2.4.4. Characterization of Average Molecular Weight

The average molecular weight of SBP-F4 was measured using DAWN Heleos II multi-angle light scattering and an Optilab T-rEX refractive index detector (MALS-RI, Wyatt Technology, Santa Barbara, CA, USA) equipped with a PL aquagel-OH MIXED-H (7.5 × 300 mm, Agilent Technologies Co., Ltd., Palo Alto, CA, USA). The mobile phase consisted of 500 mM NaCl aqueous solution at a flow rate of 0.5 mL/min. The samples (2 mg/mL) were eluted with NaCl (0.5 mol) and passed through a membrane filter with a 0.22 µm pore diameter. A refraction index increment (dn/dc) of 0.140 mL/g was used for the calculation [33]. All data collection and analysis was performed using Astra 6.1 software (Wyatt Technology Corporation, Santa Barbara, CA, USA).

#### 2.4.5. Morphological Analysis

For morphological comparison of SBP-F4, we used commercially available fucoidan isolated from brown algae *Undaria pinnatifida* (Sigma, St. Louis, MO, USA). Morphological features were recorded using a field-emission scanning electron microscope (TESCAN). SBP-F4 and commercial fucoidan were sputtered with platinum using a sputter coater prior to SEM analysis.

### 2.5. Cell Culture

The RAW 264.7 macrophage cell line was purchased from the Korea Cell Line Bank (KCLB, Seoul, Korea). RAW 264.7 cells were cultured in DMEM supplemented with 10% FBS and 1% penicillin–streptomycin. Cells were grown in a humidified incubator set at 37 °C supplemented with 5% CO_2_.

For the experiment, the Raw 264.7 cells (1.0 × 10^5^ cells/mL) were seeded in a 96-well plate for 24 h and treated with various concentrations (25, 50, 100, and 200 μg/mL) of the samples for 1 h. Next, LPS (1 μg/mL) was added to the wells and the plate was incubated for 24 h. Finally, the cell viability was determined by performing an MTT assay. Briefly, the MTT solution (10 μg/mL, 25 μL) was added to each well and incubated for 3 h. Subsequently, the formazan crystals were dissolved by adding DMSO. Finally, the amount of purple formazan was determined by measuring the absorbance at 540 nm.

### 2.6. Measurement of NO Production in RAW 264.7 Cells

Briefly, the cell culture medium (100 μL) was mixed with 100 μL Griess reagent (1% sulfanilamide and 0.1% N-(1-naphthyl) ethylenediamine dihydrochloride in 2.5% phosphoric acid) in 96-well plates. The mixture was incubated in the dark for 10 min at room temperature. The NO content in the mixture was determined by measuring the absorbance at 540 nm using a microplate reader (Synergy HT, BioTek Instruments, Winooski, VT, USA).

### 2.7. Western Blot Analysis

The effect of SBP-F4 on iNOS and COX-2 expression was assessed by Western blot analysis as described previously [34]. Briefly, RAW 264.7 cells (1.0 × 10^5^ cells/mL) were seeded in 6-well plates. After 24 h, different concentrations SBP-F4 were added to the cells and incubated for 1 h. Subsequently, LPS (1 μg/mL) was added and incubated for 24 h. After incubation, cells were washed twice with PBS. The subsequent steps were followed in line with previous research methods. A cytoplasmic extraction kit, NE-PER^®^ (Thermo Scientific, Rockford, IL, USA), was used to isolate cytosolic proteins. Proteins were standardized using a BCATM protein assay kit (Pierce, Rockford, IL, USA). Electrophoresis was carried out using 10% SDS-polyacrylamide gels. SuperSignal™ West Femto Maximum Sensitivity Substrate (Thermo, Burlington, ON, Canada) was used for the detection of protein bands on a Core Bio DavinchChemi imaging system (Seoul, Korea).

### 2.8. Measurement of Survival Rate, Cell Death, and NO Production in Zebrafish Larvae

Adult zebrafish were maintained as described previously [35]. Briefly, 10 adult zebrafish were kept in 3 L acrylic tanks maintained at 28.5 °C under a 14/10 h light/dark cycle and fed twice per day (Tetra GmbHD-49304 Melle, Germany). Subsequently, one female and two males were interbred. Embryos were collected within 30 min of natural spawning induced by light. The zebrafish experiment was approved by the Animal Care and Use Committee of Jeju National University (Approval No. 2020-0049).

Embryos were transferred to individual wells of 12-well plates containing 900 μL embryo media. At 7–9 h post fertilization (hpf) (*n* = 15), the embryos were treated with different concentrations of the samples. LPS (10 μg/mL) was added after 1 h. The groups used in the experiments were as follows: 1: control; 2: group treated only with LPS; and 3: groups treated with various concentrations of LPS. At 3 days post fertilization (dpf), zebrafish larvae were transferred to a 24-well plate and treated with acridine orange solution (7 μg/mL) for 30 min in the dark at 28.5 °C. Acridine orange is used to detect apoptotic cells [36]. Next, zebrafish larvae were rinsed twice with fresh embryo media, anesthetized with 0.003% MS-222 (tricaine methanesulfonate), and photographed under a microscope equipped with a Cool SNAP-Procolor digital camera (Olympus, Japan). Fluorescence intensity was quantified using ImageJ software. At 3 dpf, zebrafish larvae were transferred to a 24-well plate, treated with DAF-FM-DA solution (5 μM), and incubated for 3 h in the dark at 28.5 °C. After incubation, the rest of the procedure was performed as described previously.

### 2.9. Statistical Analysis

All experiments were performed in triplicate and the data are expressed as mean ± standard error (SE). One-way ANOVA was used to compare the mean values of each treatment using SPSS 12.0. Significant differences between the means of parameters were determined via Duncan’s multiple range test (* *p* < 0.05, ** *p* < 0.01, *** *p* < 0.001 compared to the LPS-treated group, ^###^ *p* < 0.001 and ^####^ *p* < 0.0001 compared to the LPS-untreated group).

## 3. Results and Discussion

### 3.1. Chemical Composition of the SBPs

**Figure 1:**
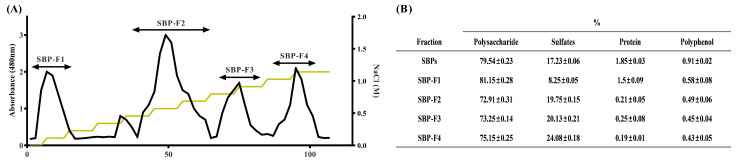
Preparation and chemical composition of SBPs and SBP fractions. (**A**) Sulfated polysaccharides purified by DEAE-cellulose anion-exchange chromatogram and (**B**) chemical composition of the total SBPs and SBP fractions.

To extract polysaccharides from *S. binderi*, we followed a previously published protocol [37]. The hydrolysate prepared with Celluclast was precipitated with ethanol, and the fractions were separated using a DEAE-cellulose column and anion-exchange chromatography. As indicated in Figure 1A, four SBP fractions (SBP-F1–4) were obtained. The chemical composition of each fraction is shown in Figure 1B. In the case of SBPs, 79.54 ± 0.23% of polysaccharides were obtained, and the sulfate content was 17.23 ± 0.06%. In the case of SBP fractions, the polysaccharide contents were 81.15 ± 0.28, 72.91 ± 0.31, 73.25 ± 0.14, and 75.15 ± 0.25% and the sulfate contents were 8.25 ± 0.05, 19.75 ± 0.15, 20.13 ± 0.21, and 24.08 ± 0.18% respectively.

The protein and polyphenol contents of SBPs and SBP fractions were lower than those of polysaccharides and sulfates (Figure 1B). In a previous study, the last fraction obtained using a DEAE-cellulose column had the highest sulfate content [30]. DEAE-cellulose is positively charged; therefore, it is commonly used to bind and isolate negatively charged substances. In a previous study, fucose-rich fractions were obtained using DEAE-cellulose [38]. Likewise, in our study, SBP-F4 contained the highest sulfate content among all the fractions. The fractions obtained by DEAE-cellulose anion-exchange chromatography were investigated using HPAE-PAD to identify the monosaccharide compositions (Table 1). The fucose content was 1.67, 1.24, 4.35, 8.00 for SBP-F1–4, respectively, and increased as expected from results presented in Figure 1B.

Marine organisms are a rich source of biologically active secondary metabolites, with potential application in pharmacological and medical fields [39]. In a previous study [40], *Saccharina-japonica*-derived polysaccharide extract containing 79.49% fucose exhibited anti-inflammatory activity in vivo and in vitro. Similarly, another sulfated polysaccharide extract obtained from *Sargassum vulgare,* which contained up to 50% fucose, showed antioxidant and anti-inflammatory effects [41]. These results show that fucose-containing sulfated polysaccharides exert beneficial bioactivity and are associated with antioxidant activity and anti-inflammatory activity.

Some reports have shown that the content of sulfated polysaccharides generally promotes anti-inflammatory activity, although the reason underlying this effect has not yet been clarified. Various sulfated polysaccharides have shown direct anti-inflammatory activity by suppressing the expression of inducible nitric oxide synthase (iNOS) in LPS-stimulated RAW264.7 macrophages and preventing excessive NO production [42]. In addition, COX-2 enzyme, a factor regulating platelet accumulation, vascular permeability, and thrombogenesis during inflammation, is significantly inhibited by sulfated polysaccharides [43]. Thus, fucose-containing polysaccharides are causally related to anti-inflammatory activity; thus, SBP fractions separated by DEAE column are expected to have inflammation-inhibitory effects.

### 3.2. FTIR Spectrum of Sulfated Polysaccharides

**Figure 2:**
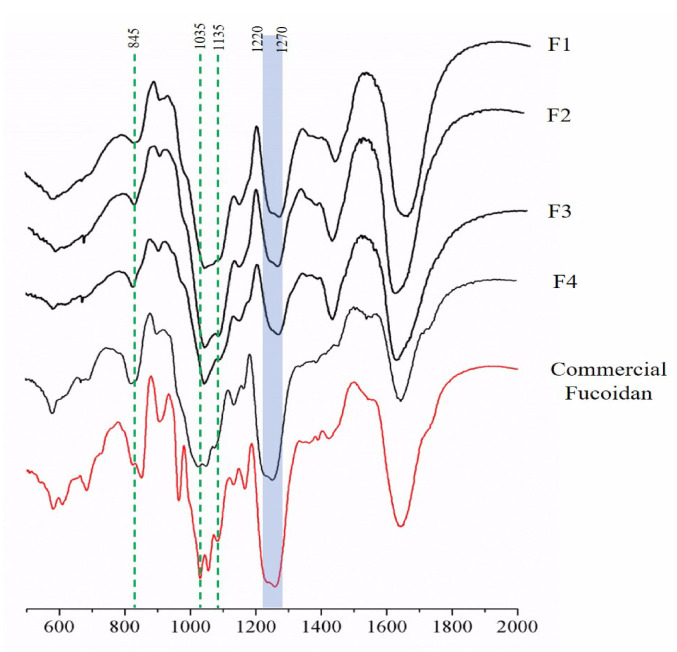
FTIR spectra of SBP-F1–4.

To characterize the structural properties of polysaccharides, we used the 400–2000 cm^−1^ wave number range of the FTIR spectra covering the fingerprint region of polysaccharides. The FTIR spectra showed prominent peaks at 845, 1035, and 1616 cm^−1^, and a broad peak between 1220 and 1270 cm^−1^ in the SBP fractions and the commercial fucoidan (Figure 2). Glycoside bonds between multiple monomers to form polysaccharides were observed at 1000–1100 cm^−1^ (C–O–C stretching vibrations). The strong absorption band at 1220–1270 cm^−1^ confirmed the significant amount of sulfate in the fractions and commercial fucoidan. For each fraction, the intensities of most peaks were similar in all spectra, but a definite difference was observed for the intensity of 1220–1270 cm^−1^. The peak identified at 1200 cm^−1^ corresponds to sulfate stretching vibrations [44], and the results suggest that sulfate-content-wise, SBP-F4 has a strong similarity to commercial fucoidan.

### 3.3. Anti-Inflammatory Effect of Sulfated Polysaccharides on LPS-Activated RAW 264.7

**Figure 3:**
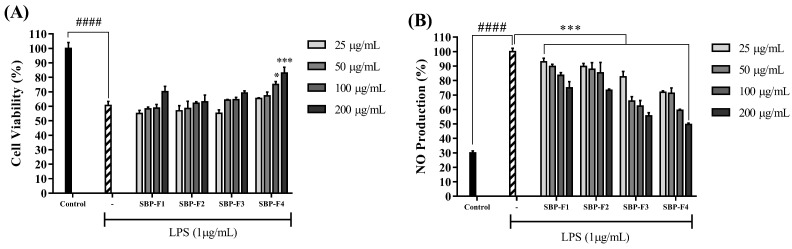
Effect of SBP fractions on RAW 264.7 cell viability (**A**) and NO production (**B**). * *p* < 0.05, *** *p* < 0.001 compared to the LPS-treated group, #### *p* < 0.0001 compared to the control group.

**Figure 4:**
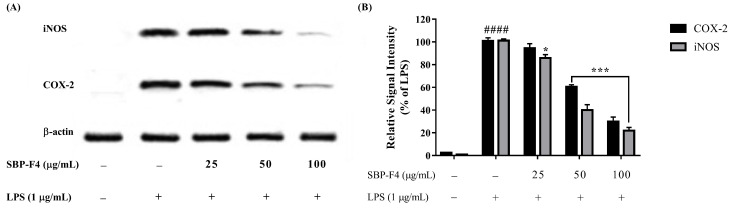
The inhibitory effects of SBP-F4 on LPS-activated inflammation-associated protein expression in RAW 264.7 cells. (**A**) Protein expression of iNOS and COX-2 were measured with Western blot, (**B**) Quantitation of Western blot-band of COX-2 and iNOS in LPS-treated group with SBP-F4. Data expressed as mean ± SE. * *p* < 0.05 and *** *p* < 0.001 as compared to LPS-treated group and #### *p* < 0.0001 as compared to control group.

LPS induces NO production by stimulating inflammatory responses in macrophages. As illustrated in Figure 3A, the production of NO decreased in a dose-dependent manner upon treatment with SBP fractions. The fractions close to F4 exhibited inflammation-inhibitory activity, and cell viability increased in response to co-treatment with LPS and SBP fractions (Figure 3B). The inhibition of iNOS and COX-2 proteins by SBP-F4 was evaluated by Western blot analysis (Figure 4A). The iNOS and COX-2 protein expression levels decreased in a dose-dependent manner in response to SBP-F4 treatment (Figure 4B). Hence, these results clearly show the anti-inflammatory potential of SBP-F4.

### 3.4. Anti-Inflammatory Effect of Sulfated Polysaccharide on LPS-Treated Zebrafish Larvae

**Figure 5:**
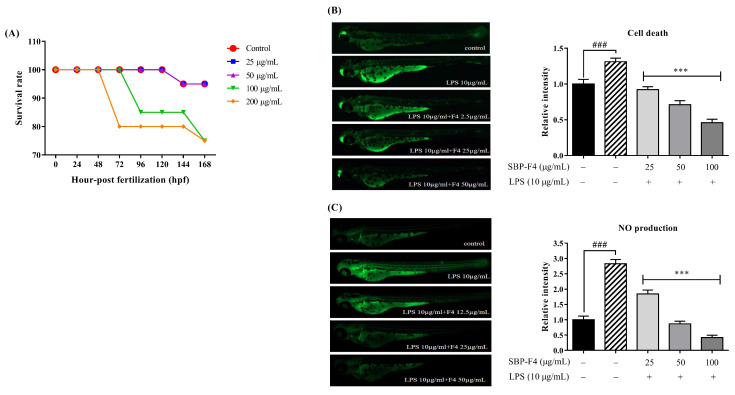
Effect of SBP-F4 on survival rate, LPS-induced cell death, and NO production in zebrafish larvae. (**A**) After fertilization (7–9 h post fertilization (hpf)), zebrafish embryos were moved to a 12-well plate. The embryos were pretreated with various concentrations of SBP-F4 (25, 50, 100, and 200 µg/mL) and compared to the untreated control group. The survival rate of zebrafish larvae was 168 hpf after treatment. Inhibitory effects of SBP-F4 on LPS-induced cell death (**B**) and NO production (**C**) in zebrafish larvae. The fluorescence intensity of individual zebrafish larvae were quantified using ImageJ software. Experiments were performed in triplicate; data are expressed as the mean ± SE. *p* < 0.05 and *** *p* < 0.001 compared to the LPS-treated group (10 µg/mL) and ### *p* < 0.001 compared to the control group (nontreated SBP-F4 and LPS).

We first tested the toxicity of SBP-F4 using an in vivo model, by determining survival rates after exposing zebrafish larvae to 25, 50, 100, and 200 µg/mL of SBP-F4 and comparing the outcomes to those of nontreated control zebrafish larvae (Figure 5A) as described previously [45]. The results show that SBP-F4 at concentrations up to 100 µg/mL had no significant toxic effects. Hence, we selected SBP-F4 concentrations of 25, 50, and 100 µg/mL for the subsequent zebrafish larvae experiments. Next, to evaluate the protective effect of SBP-F4 on LPS-induced cell death in zebrafish larvae (3 dpf), acridine orange fluorescence intensity was measured. Zebrafish larvae treatment with SBP-F4 at 25, 50, and 100 μg/mL significantly reduced LPS-induced cell death in zebrafish larvae, while the zebrafish larvae that were treated with only LPS showed a significant increase in cell death (Figure 5B). Following this, the DAF-FM-DA intensity was measured to evaluate the NO production in SBP-F4-treated zebrafish larvae. As observed with cell death, NO production was significantly increased in LPS-treated zebrafish larvae, but decreased in a dose-dependent manner in SBP-F4-treated zebrafish larvae (Figure 5C).

### 3.5. Characterization of SBP-F4

MALS is used to check molar mass, polydispersity, and rms radius [46]. We found that SBP-F4 had a number-average molecular weight (Mn) of 1.986 × 10^5^ g/mol, a weight average molecular weight (Mw) of 2.351 × 10^5^ g/mol, and a Z-average molecular weight (Mz) of 2.867 × 10^5^ g/mol. Moreover, LC/MS analysis in negative-ion mode of SBP-F4 revealed the Fuc at m/z 163.35 as the main component together with ions of GalS(GalSO_3_) at m/z 259.36 and (Fuc)_2_+SO_3_−H_2_O at m/z 389.15 (Supplementary Figure S1A).

**Figure 6:**
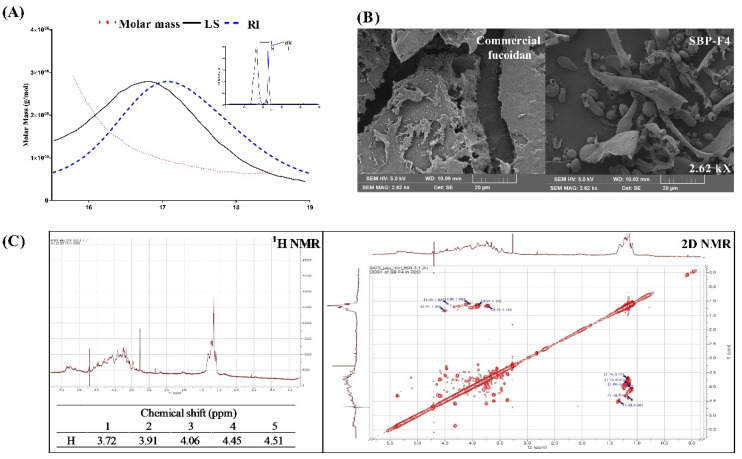
Identification and characterization of SBP-F4. (**A**) GPC-RI-MALS profile of SBP-F4, (**B**) morphological properties of commercial fucoidan and SBP-F4 as identified using SEM, and (**C**) ^1^H and 2D NMR spectra of SBP-F4.

SEM was performed to compare the surface of SBP-F4 with the commercially available fucoidan. At 2.62 k× magnification, when looking at the relatively wide range of particle distribution between commercial fucoidan and SBP-F4, we observed that SBP-F4 was homogeneously thinner than the commercial fucoidan (Figure 6B). Furthermore, SBP-F4 pores were found to be more distinct than that of the commercial fucoidan.

To better clarify the structural characteristics of commercial fucoidan and SBP-F4, ^1^H NMR spectroscopy was performed. According to the ^1^H NMR spectra, commercial fucoidan and SBP-F4 contain sugar residues at H1–H5 (proton signals from 3.30–4.60 ppm) [47]. To perform a detailed characterization of the total H chemical shifts, 2D NMR spectra were generated as shown in Figure 6C. Based on the 2D NMR spectrum, the chemical shifts of H1–H5 were assigned based on the COSY spectra. The proton signal from 5.20 to 5.40 ppm was α-anomeric and indicates a region rich in alpha-D-linked polysaccharide [48]. The signals at the region from 1.0 to 1.5 ppm represent methyl groups bound to fucopyranose [49].

## 4. Conclusions

In conclusion, sulfated polysaccharides containing sulfated fucose as the major monosaccharide, were purified from the brown algae *S. binderi* using DEAE-cellulose anion-exchange chromatography. These polysaccharides were structurally evaluated using FTIR, NMR, and MALS. The results showed that SBP-F4 had the highest fucose content among the SBP fractions and a strong sulfate stretching vibration comparable to that of commercially available fucoidan. Results of in vitro and in vivo studies confirmed the biological activities of SBPs, as SBP-F4 treatment inhibited cytotoxicity and NO production in LPS-activated macrophages and zebrafish larvae. Moreover, SBP-4 treatment significantly inhibited COX-2 and iNOS protein levels in LPS-activated macrophages and reduced cell death and NO production in LPS-treated zebrafish larvae. Therefore, sulfated polysaccharides derived from *S. binderi* can be utilized as potential anti-inflammatory agents in the future.

## Figures and Tables

**Table 1:** Monosaccharide composition analysis via HPAE-PAD spectrum.

Monosaccharide composition (molar ratio)		SBP-F1	SBP-F2	SBP-F3	SBP-F4
	Fucose	1.67	1.24	4.35	8.00
	Galactose	1.25	1.34	5.84	7.61
	Glucose	1	1	1	1
	Mannose	0.98	1.56	2.51	N.D.
	Arabinose	N.D.	0.17	0.40	0.19
	Rhamnose	0.37	0.41	0.52	0.30

Note: the molar content of glucose was set as “1” to calculate the content of other monosaccharides.

## Data Availability

All data are contained in article and Supplementary Material.

## Supplementary Materials

The following are available online at https://www.mdpi.com/article/10.3390/antiox10060822/s1, Figure S1: Negative-ion mode ESIMS of SBP-F4.
