# Supplementary material for: Purification and Structural Characterization of Sulfated Polysaccharides Derived from Brown Algae, Sargassum binderi: Inhibitory Mechanism of iNOS and COX-2 Pathway Interaction
Source: Antioxidants (Basel). 2021 May 21;10(6):822. doi: 10.3390/antiox10060822 (PMC8223978; doi:10.3390/antiox10060822)
Supplement: Supplementary file 1 [file antioxidants-10-00822-s001.zip › antioxidants-1182200-SI.pdf]

In supplementary data:

Materials and Methods

ESI-MS analysis

SBP-F4 (5 mg) was placed in a 20-mL vial and treated with 0.5 mL of 2 M trifluoroacetic acid (TFA) for 8 h at 100 °C in a dry oven. Acid-hydrolyzed SBP-F4 was analyzed using high-performance liquid chromatography/mass spectrometry (HPLC/MS). HPLC analysis employed a Waters HPLC system with mobile phase consisting of water-acetonitrile (ACN) using a gradient method (0 min 10:90 v/v; 0–40 min 10:90–50:50 v/v; 40–41 min 10:90 v/v; 41–55 min 10:90 v/v). InfinityLab Poroshell 120 Hilic-z (2.7 4.6100 mm column, Agilent, Santa Clara, CA, USA) column used was, with a flow rate of 0.5 mL/min.

ESI-MS spectra were generated using an ACQUITY QDa mass spectrometer (Waters) with a dual electrospray ionization source. All spectra were acquired in negative-ion mode. The capillary voltage was set to 3.0 kV, and the cone voltage to 40 V. The collision energy was optimized as low 6 eV/high CE 15–45 eV. The source and desolvation temperatures were 100 °C and 30 °C, respectively. The cone gas flow was 50 L/h, and the desolvation gas flow was 800 L/h.

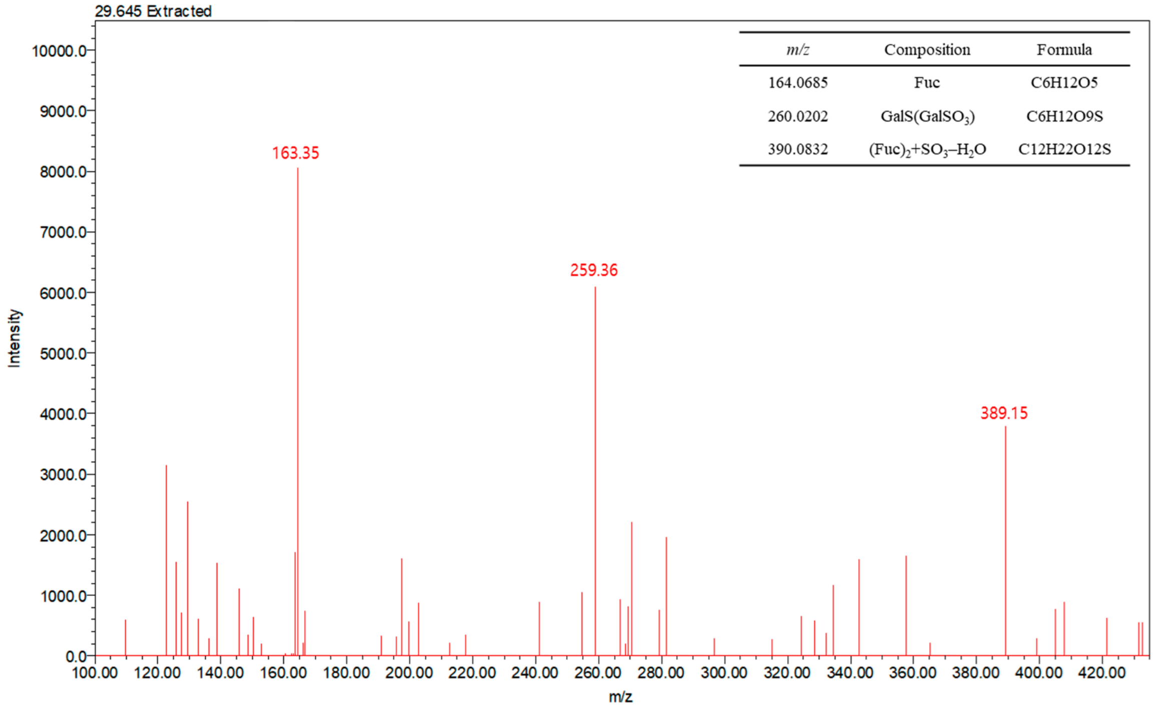

Figure S1. Negative-ion mode ESIMS of SBP-F4.
